# Under utilisation of the 2-week wait initiative for lung cancer by primary care and its effect on the urgent referral pathway

**DOI:** 10.1038/sj.bjc.6602798

**Published:** 2005-09-27

**Authors:** N R Lewis, I Le Jeune, D R Baldwin

**Affiliations:** 1 Department of Respiratory Medicine, David Evans Centre, Nottingham City Hospital, Hucknall Road, Nottingham NG5 1PB, UK

**Keywords:** lung cancer, waiting times, diagnosis, investigation, symptoms

## Abstract

The ‘2-week wait’ scheme for referral of patients with cancer to secondary care coincided with the introduction of Department of Health (DoH) Guidelines on referral of patients with suspected lung cancer. The aim of this study was to examine the impact of this process on the urgent referral pathway for lung cancer. Medical records of all patients referred with suspected lung cancer were reviewed for the year prior to introduction of the 2-week wait and DoH guidelines and for the subsequent 24 months. A total of 1044 patients were referred, of which 650 (62%) were found to have malignancy. In the first and second years of the 2-week wait scheme, only 57 and 58% were referred via the scheme. Department of Health guidelines were followed in all but a small number. Median wait time increased from 7 to 9 days. The proportion of all urgent referrals seen within 2 weeks fell from 84 to 71%. The proportion of non-2-week wait urgent referrals being seen within 2 weeks was only 75.5% in the first year of the scheme and fell further to 60.9% in the second year. The absolute number of referrals rose and the proportion having cancer fell from 78% before the scheme to 46% in the second year. During this time, there was no change in stage at presentation. Symptoms were not helpful in discriminating benign from malignant disease and haemoptysis was actually more common in the benign group. However, over 50% of patients in the benign group were appropriate to be seen in secondary care. The 2-week wait scheme has so far failed to reduced waiting times for lung cancer. The findings of this study suggest that this is partly due to continued usage of urgent referral routes outside the 2-week wait scheme and secondly due to a large increase in referrals, probably generated by the introduction of the DoH guidelines. Some adjustment to the guidelines may be appropriate to reflect more emphasis on the early performance of a chest X-ray and the use of direct access to other imaging modalities such as CT. Patients referred outside the 2-week wait are disadvantaged and thus practitioners would be wise to refer all their patients through the 2-week wait system.

The Government white paper *The New NHS – Modern, Dependable* (Department of Health National Standards, Local Action, 2004) proposed that everyone with suspected cancer should be given the opportunity to be seen by a specialist within 2 weeks of the date of the GP referral. In order to introduce this ‘2-week wait’ principle to lung cancer, the Department of Health (DoH) introduced national guidelines for the urgent referral of suspected lung carcinoma in April 2000. The intention was to simplify and streamline the referral process from primary care to respiratory physicians, thus expediting treatment by specialist multidisciplinary units (Department of Health NHS Improvement Plan June, 2004).

Here, we report on the utilisation and impact of this process on the urgent referral pathway for lung cancer.

## MATERIALS AND METHODS

Medical records of all patients referred with suspected lung cancer to Nottingham City Hospital in the 12 months prior to the introduction of DoH guidelines (1st April 1999–31st March 2000) and the subsequent 24 months (1st April 2000–31st March 2002) were retrospectively audited by three observers (NRL, DRB, ILJ). The reason for referral, referral route and times from GP referral to first hospital visit, investigations, diagnosis and treatment were recorded along with outcome data including surgical stage histological diagnosis, management plan and treatment modality. Statistical comparisons for these nonparametric data were made using the Mann–Whitney *U*-test. Fisher's exact test and *χ*^2^ tests were used for comparisons of proportions.

## RESULTS

### Number of patients referred and referral route

A total of 1044 patients were referred to the respiratory physicians with a suspected diagnosis of lung cancer during the 3-year audit period. In all, 628 (60.2%) were male and the median age was 73 years (range 24–98 years). Malignancy was confirmed in 650 (62.3%), of which 578 (88.9%) were found to have a primary lung tumour. Of these 465 (80.4%) were non-small-cell carcinoma, 86 (14.9%) small-cell carcinoma, 22 (3.8%) mesothelioma and five (0.9%) were other tumours.

Table 1 shows the number of referrals for each year. Only 57% of patients were referred via the 2-week wait scheme (201/352) in 2000/2001 and 58% (236/404) in 2001/2002.

### Adherence to DoH guidelines for referral

Only four patients (1% of referrals) in the first year after introduction of the guidelines, and 16 patients (4% of referrals) in the second year failed to meet the criteria for urgent referral listed in the DoH guidelines.

### Effect of introduction of 2-week wait and DoH guidelines on waiting times

Table 2 shows the median waiting times to points on the patient pathway. Introduction of the 2-week wait principle and the DoH guidelines for urgent referral has resulted in patients waiting longer for their first hospital visit (median of 7 *vs* 9 days) and longer for treatment of lung cancer. Time to diagnosis initially increased and then returned to preguideline values.

The proportion of patients who were seen within 2 weeks was 84% prior to the introduction of the ‘2-week wait’ scheme and 85% in the year after introduction of the 2-week wait. The proportion fell to 71% (*P*<0.0001; Mann–Whitney *U*-test) in the year 2001–2002. Analysis of the median waiting time by referral route demonstrated that all of those patients referred via the ‘2-week wait’ scheme were seen within that time; however, as a result of the increase in absolute urgent referral numbers, the percentage of those referred by a conventional route who were seen within 2 weeks dropped significantly to 75.5% in 2000–2001 and further to 60.9% (*P*<0.0001) in 2001–2002.

### Reasons for referral

The most common reasons for referral were cough, dyspnoea, weight loss, haemoptysis and chest pain. Table 3 shows the proportion of patients with each symptom by year, divided into patients with and without cancer. There was no difference in the pattern of symptoms after introduction of DoH guidelines. The frequency of these symptoms was found to be high in both patients with and without cancer. Chest pain, dyspnoea and weight loss were all more common in patients with lung cancer (*P*<0.0001), but were still present in a large enough proportion of patients without cancer to mean they were not good discriminators. Haemoptysis was significantly more common in benign diagnoses (*P*<0.0001).

An abnormal chest X-ray suggestive of lung cancer was the most common referral basis accounting for 87.2% over the 3-year period. The finding of a mass was more common in cancer (*P*<0.0001), but 30% of benign diagnoses also had a mass on chest radiograph. Consolidation and interstitial shadowing were more common in nonmalignant conditions (*P*<0.0001).

### Proportion of patients having cancer

Table 4 shows the final diagnosis in all referrals. The proportion of urgent referrals subsequently found to have a malignancy decreased from 78% (224/286) prior to the introduction of the guidelines and the ‘2-week wait’ scheme, to 68% (240/352) of patients in the first year (*P*<0.0001) and 46% (186/404) of patients in the second year after their introduction (*P*<0.0001).

### Stage of non-small-cell lung cancer

Table 5 shows the number of patients in each surgical stage, where stage was recorded. In 1999–2000, 75% of patients were staged, in 2000–2001, 71% and in 2001–2002, 77%. There was no significant change in stage (*χ*^2^, 2.52; *P*=0.47)

### Benign diagnoses

Table 6 shows the diagnostic categories for benign conditions referred as suspected lung cancer. Infections represented the largest increase in benign diagnoses. These cases were thought appropriate to refer to secondary care in 56, 54 and 51% of patients in years 1999–2000, 2000–2001 and 2001–2002, respectively. In many other cases, however, it may have been appropriate on medical grounds alone to expedite a diagnosis by urgent referral, either because the condition required treatment or there was a high probability that there might be cancer.

## DISCUSSION

Introduction of the target ‘2-week wait’ scheme and DoH guidelines on urgent referral of suspected lung cancer has so far failed to achieve its primary objective of reducing waiting times. The data generated by this paper suggest two linked explanations for this unexpected finding: firstly, despite the publication of national guidelines recommending urgent referral through the ‘2-week wait’ scheme and local efforts to encourage this by simplifying referral procedures with proforma-based faxed referral letters/electronic referrals, a significant proportion of patients continued to be referred by standard means. Only 57.5% of patients were referred using the recommended ‘2-week wait’ scheme. 37.7% were referred urgently via conventional means.

Secondly, there has been an increase of 42% in the number of urgent referrals for suspected lung cancer. This may reflect increased awareness of the problem in primary care, the perception of improved access to specialist teams or the inflexibility resulting from specific guidelines. The latter is suggested by the clear implication in some of the referral letters of doubt about whether the patient should be seen by a specialist. There may also have been a tendency to abuse the rapid access to specialist advice, although most patients still fitted DoH criteria. All of these factors may have resulted in the finding that the proportion of referrals culminating in malignant diagnoses has decreased from 78 to 46% in the 2 years since the guidelines were implemented.

These linked observations (more referrals with lower proportion of malignancy and a significant proportion of patients referred outside the 2-week wait system) have two important consequences: firstly that assessment of increasing numbers of patients with benign disease will place further strain on the lung cancer services, and secondly that the patients referred outside the 2-week wait will be disadvantaged.

The first of these consequences is demonstrated in this paper by the increase in median time to first clinic visit from 7 to 9 days despite improvements in the efficiency of the referral process (including the appointment of an additional consultant). The second is illustrated by the finding that the waiting times were longer for non-2-week wait referrals. The increased number of referrals may have a beneficial influence on earlier diagnosis of lung cancer, but this was not supported by the findings of this study, as the stage did not change significantly. Moreover, making an earlier diagnosis may improve survival but not necessarily mortality – an effect of lead-time bias.

Thus, introduction of the guidelines and target wait scheme has had a beneficial effect for those patients referred by the recommended route, but a negative effect on those referred urgently outside this system. Furthermore, waiting times reported to the Government are based on patients referred through the 2-week wait system and thus Government figures on waiting times for lung cancer do not reflect the true statistics. This may also apply to other cancers. Although out-with the scope of this paper, these data do prompt speculation that waiting times for urgent and routine noncancer referrals seen by the same consultants might also have increased as a consequence of the rapid increase in suspected cancer patients referred since the introduction of the guidelines.

The observed increase in early review of patients without cancer does have beneficial effects. The reassurance provided by rapid diagnosis and removal of a lengthy period of uncertainty is important for the individual. Furthermore, the study showed that over 50% of patients without cancer should probably have been in a respiratory medicine clinic in any case irrespective of cancer referral guidelines. Thus, the extra resources have had other ancillary benefits.

Despite the benefits of seeing more patients quickly, if the proposed Government targets of reducing the delay from referral to treatment to no more than 2 months by December 2005 (NHS Executive, 2000; National Institute for Clinical Excellence, 2005) are to be achieved, it is important that accurate baseline statistics are recorded. If the increase in referrals continues, considerable further investment in cancer services will be required and referral guidelines may need to be modified to reflect the management of minor haemoptysis. Currently, NICE (NICE, 2005) recommend offering the patient a chest radiograph prior to referral in patients presenting with haemoptysis. So far, it cannot be recommended that such patients are followed with a simple radiograph, but direct access to CT scanning and possibly bronchoscopy could reduce the burden on outpatient appointments while still identifying those patients with benign disease who may need a respiratory physician. Lastly, general practitioners should be aware that not using the 2-week wait system gives inaccurate national waiting time data and more importantly may disadvantage their patients.

## Figures and Tables

**Table 1:** Number of referrals of suspected lung cancer

	**1st April 1999–31st March 2000, *n* (%)**	**1st April 2000–31st March 2001, *n* (%)**	**1st April 2001–31st March 2002, *n* (%)**
Total number of referrals	286	352	404
			
2-Week wait referral	2 (0.6)	201 (57)	236 (58)
Urgent referral	279 (97.5)	142 (40)	153 (38)
Other referral^a^	5 (1.7)	9 (2.5)	17 (4.2)

a Patients who were found to have lung cancer but were not referred via a recognised urgent route.

**Table 2:** Effect of introduction of 2-week wait and DoH guidelines on waiting times

	**Waiting time in days: median (range)**			
	**1999–2000 (Department of Health National Standards, Local Action, 2004)**	**2000–2001 (Department of Health NHS Improvement Plan June, 2004)**	**2001–2002 (National Institute for Clinical Excellence, 2005)**	***P*-value**
Referral to first hospital visit	7 (0–124)	8 (0–101)	9 (0–98)	0.0009 for 1 *vs* 3
Referral to diagnosis	26 (0–228)	33 (2–307)	27 (0–300)	<0.00001 for 1 *vs* 2; 0.0003 for 2 *vs* 3
First hospital visit to diagnosis	15 (0–219)	21 (0–294)	15 (0–300)	<0.00001 for 1 *vs* 2 and 2 *vs* 3
Referral to treatment	37 (2–228)	41 (2–307)	42 (0–239)	<0.04 1 *vs* 2 or 3

DoH=Department of Health.

**Table 3:** Predominant symptoms of patients with and without cancer

	**Year**											
	**April 99–March 2000**				**April 2000–March 2001**				**April 2001–March 2002**			
	**Cancer**		**Benign**		**Cancer**		**Benign**		**Cancer**		**Benign**	
	**Absolute**	**%**	**Absolute**	**%**	**Absolute**	**%**	**Absolute**	**%**	**Absolute**	**%**	**Absolute**	**%**
Total	224	100.0	62	100.0	240	100.0	112	100.0	186	100.0	218	100.0
												
Cough	76	33.9	40	64.5	89	37.1	88	78.6	101	54.3	127	58.3
Dyspnoea	131	58.5	23	37.1	131	54.6	59	52.7	93	50.0	71	32.6
Weight loss	102	45.5	16	25.8	151	62.9	40	35.7	55	29.6	44	20.2
Haemoptysis	52	23.2	19	30.6	45	18.8	34	30.4	43	23.1	66	30.3
Chest pain	97	43.3	10	16.1	116	48.3	40	35.7	43	23.1	30	13.8

**Table 4:** Final diagnosis in all urgent referrals

	**Year**					
	**April 99– March 2000**		**April 2000– March 2001**		**April 2001– March 2002**	
	**Absolute**	**%**	**Absolute**	**%**	**Absolute**	**%**
Total referrals	286	100.0	352	100.0	404	100.0
						
All cancers	224	78.3	240	68.2	186	46.0
Primary lung cancers	202	70.6	212	60.2	164	40.6
NSCLC	161	56.3	173	49.1	131	32.4
SCLC	37	12.9	30	8.5	19	4.7
Mesothelioma	3	1.0	8	2.3	11	2.7
Carcinoid	1	0.3	1	0.3	3	0.7
Lymphoma	6	2.1	3	0.9	3	0.7
Metastatic	16	5.6	25	7.1	18	4.5
No cancer	62	21.7	112	31.8	218	54.0

NSCLC=non-small-cell carcinoma; SCLC=small-cell carcinoma.

**Table 5:** Stage of non-small-cell lung cancer

	**Stages**			
**Year (s)**	**1**	**2**	**3**	**4**
1999–2000	32	8	40	34
2000–2002	48	23	79	54

*χ*^2^=2.51; *P*=0.47.

**Table 6:** Benign diagnoses in patients referred urgently for suspected lung cancer

	**Frequency**		
**Condition**	**1999–2000**	**2000–2001**	**2001–2002**
*Conditions that can usually be managed in primary care*			
Infection	18	41	83
COPD	3	3	7
Goitre	3	1	0
Normal variant	1	1	9
ENT cause	1	3	5
CCF	1	2	2
			
*Conditions usually managed by respiratory physicians (or complex)*			
Vascular markings	3	4	3
TB (old or active)	3	7	4
Benign lung tumour	1	0	5
Benign pleural shadows	5	6	17
Sarcoidosis	1	3	2
Benign lung shadow	5	0	15
Interstitial lung disease	4	19	9
Bronchiectasis	3	9	13
Other	0	4	13
DNA, died or refused tests	4	6	10
No diagnosis	6	3	21
			
Total	62	112	218

COPD=chronic obstructive pulmonary disease; TB=tuberculosis.
